# Effective Methods of Restraining Diffusion in Terms of Epidemic Dynamics

**DOI:** 10.1038/s41598-017-06171-6

**Published:** 2017-07-20

**Authors:** Fei Xiong, Zhao-Yi Li

**Affiliations:** 1 School of Electronic and Information Engineering, Beijing Jiaotong University, Beijing, 100044 China; 2 Key Laboratory of Communication and Information Systems (Beijing Jiaotong University), Beijing Municipal Commission of Education, Beijing, 100044 China

## Abstract

Removing influential nodes or shortcuts in a network restrains epidemic or information diffusion, but this method destroys the connectivity of the network and changes the topological structure. As an alternative, an additional field can be imposed in the network to affect node behaviors and slow down diffusion dynamics. However, little research has been performed systematically to analyze and compare these methods. This paper investigates epidemic dynamics and proposes the following four methods to restrain the diffusion process: blocking nodes, blocking edges, distracting node attention, and propagating opposite information. We compare differences in the actions of these methods, and investigate their joint effects. Through numerical experiments in a scale-free network and a real network, we observe that these methods change the spreading threshold and final extent with different conditions. The method of blocking nodes is more efficient and economical than blocking edges. Propagating opposite information can effectively prevent diffusion of target information that has a large spreading rate, whereas distracting node attention only takes effect for the information with a small rate. Meanwhile, the effects of these two methods mainly depend on their action time. From the joint effects, we can select the optimal method for different situations.

## Introduction

There is currently considerable research on modeling and analyzing dissemination dynamics. Diffusion models capture the features of microscopic interactions, and determine the rules of node behaviors^1, 2^. Approaches from statistical physics, applied probability, graph theory and other methodologies are applied to determine the spreading threshold, relaxation time and other characteristics^3–5^. Early studies investigated the propagation of epidemic diseases over an entire population. Epidemic models include the susceptible - infected (SI) model^6, 7^, the susceptible- infected - susceptible (SIS) model^8^, and the susceptible - infected - refractory (SIR) model^9–11^. In the SIR model, the cessation of diffusion behaviors is caused by spontaneous node immunization. The rumor model describes the diffusion process of rumors among people, and nodes stop propagating when they contact other spreaders or refractory nodes^12–14^. These models and their revised versions are also used to characterize information diffusion^15, 16^. The forgetting mechanism was introduced to the rumor model, which describes the diffusion process in a real network called LiveJournal. The results demonstrated that when the average network degree is sufficiently large, the influence of a rumor reaches saturation^17^. Information diffusion in online social networks was explored, and reposting behaviors were considered among node interactions^18^. Meanwhile, the addition of a behavioral classification mode into an existing epidemic framework revealed the impact of available information on the population^19^. In addition to these methods, an evolutionary game theoretic framework was also used to model diffusion dynamics, and heterogeneous actions and socio-economic interactions among nodes were elaborated in the model^20^. The availability of diffusion models was validated over real networks, and the topological structure of the underlying network seriously affected the dynamic process. An experiment was conducted to study the effect of network structure on behavior diffusion, demonstrating that behaviors spread farther and faster across clustered-lattice networks than across random networks^21^. For information diffusion in a network of microblogs, scale-free degree distribution achieves optimal diffusion dynamics^22^. When social-physical networks are conjoined, information diffusion is further accelerated based on the SIR model^23^.

For harmful epidemics or information, it is valuable to determine strategies that can effectively prevent diffusion. Many studies concentrate on detecting influential nodes^24^ or community bridges in a network and then remove them to prevent information diffusion^25^. Both node isolation and quarantine were included in epidemic dynamics according to self-compliance with the medical requests^26^. In the context of disease spreading, coreness constitutes a better topological descriptor to identify influential spreaders^27^. However, for rumor dynamics, coreness does not determine the spreading capabilities of nodes, but it can be used to prevent the diffusion of a rumor to a system-wide scale^28^. Analogously, weak ties in online social networks have a similar effect, and they might be of use in the control of information diffusion^29^. In ref. 30, an efficient network immunization strategy was put forward, and the network was partitioned into clusters with an approximately equal size. This graph-based strategy requires fewer immunization doses. Moreover, critical blocks were defined as sets of nodes that evenly partition the entire network at a small cost^31^. Critical blocks play a key role during the diffusion process. These strategies require global knowledge of the network. When network knowledge is not available, the immunization of random acquaintances of random nodes is efficient for networks of any broad-degree distribution^32^.

In many studies, the method of removing nodes is adopted to restrain the diffusion, but network connectivity is also reduced by this method. In addition to changing the topological structure of the network, one can select an alternative method to achieve the same goal, such as imposing a field into the system to affect node behaviors. These methods have rarely been investigated and systematically compared. The joint effects of integration of these methods are worth further exploration. Furthermore, the effects of these methods are different for information with different spreading capabilities and for different action time. It is unclear how to find a practical and economical method to effectively prevent diffusion in different situations. For instance, if the information is checked in the initial stage or after the early stage of the diffusion, which method is suitable to restrain it? In this paper, we present four methods to restrain information diffusion in static networks. These methods can be divided into two categories: altering the network structure, and imposing an addition field. We compare the outcomes of these methods via analysis and numerical simulations. The results show that these methods have different control capacities for diffusion, and they change the final diffusion scale and spreading threshold to different degrees. We also investigate coactions and dependence on attributes of target information, and provide an appropriate method for different situations.

The rest of this paper is structured as follows. Section 2 briefly reviews the traditional SIR model to describe information diffusion, and provides relative notations of macroscopic states. Section 3 proposes four restraining methods, and analysis is conducted. Section 4 presents numerical simulations and discussions about these methods. Concluding remarks are presented in Section 5.

## Epidemic Model

The SIR model^33^ was originally used to describe the diffusion of epidemic diseases. The model^23^ and its modified versions^17, 18^ were also applied in information diffusion. In terms of epidemic models, nodes spread information to their neighbors, and information diffusion is characterized by a stochastic process. Nodes may be in one of the following three possible epidemic states: the susceptible state, the infected state and the refractory state. The susceptible state means that nodes are unaware of the information. When nodes are affected by neighbor’s information, they become a spreader and enter the infected state. Nodes in the refractory state lose interest in the information and will never be infected again. The model is governed by the following rules^33^.

- When a susceptible node meets an infected node, the susceptible node is infected with probability *λ*.
- Each time, infected nodes become refractory with probability *δ*.

At the beginning of the dynamics, a node is infected, acting as the initial spreader, whereas all the other nodes are susceptible. The diffusion process continues until all infected nodes become refractory.

As the underlying network may be heterogeneous, nodes with different degrees should be investigated separately. In this paper, the underlying network is a static network. It is assumed that node degrees follow distribution *P*(*k*). The probability *P*(*k*′|*k*) represents degree correlations among nodes. The overall densities of nodes at degree *k* in the susceptible, infected and refractory states are defined as *ρ*_*s*_(*k*, *t*), *ρ*_*i*_(*k*, *t*), and *ρ*_*r*_(*k*, *t*), respectively. The transition of these densities is listed as follows.

1 $$\begin{array}{c}\frac{\partial {\rho }_{s}(k,t)}{\partial t}=-{\lambda }k{\rho }_{s}(k,t)\sum _{k^{\prime} =0}^{+\infty }P(k^{\prime} |k){\rho }_{i}(k^{\prime} ,t)\\ \frac{\partial {\rho }_{i}(k,t)}{\partial t}={\lambda }k{\rho }_{s}(k,t)\sum _{k^{\prime} =0}^{+\infty }P(k^{\prime} |k){\rho }_{i}(k^{\prime} ,t)-\delta {\rho }_{i}(k,t)\\ \frac{\partial {\rho }_{r}(k,t)}{\partial t}=\delta {\rho }_{i}(k,t)\end{array}$$

In uncorrelated networks, the degree-degree correlations can be written as

2 $$P(k^{\prime} |k)=k^{\prime} P(k^{\prime} )/\bar{k}$$

where $\bar{k}$ indicates the average degree of the network. Then, from Eq. (1), one obtains the spreading threshold of the model below which information cannot propagate in the network.

3 $$\lambda  > \frac{\bar{k}}{\overline{{k}^{2}}}\delta $$

## Methods

### Block diffusion at important nodes

We can block certain important nodes from participating in the diffusion. If a node is blocked, it will never be infected by neighboring spreaders, and information cannot traverse it. If we block influential nodes that play a significant role in the diffusion, we may prevent the information from spreading beyond a local region or reduce the diffusion scale. We block some influential nodes before the diffusion process. The influence of nodes may be reflected by their status in the topological structure. The indicators to describe node influence are listed as follows.

1. Node degree
  Nodes with a large degree spread information to more neighbors. If they are blocked, the chance that susceptible nodes contact the information is reduced. With this method, nodes are sorted according to their degrees, and a proportion *γ* of nodes at the top of the list will be blocked.
  Now, we consider a special case in which an uncorrelated scale-free network mediates the diffusion. In a scale-free network, it is satisfied that $\overline{{k}^{2}}={\bar{k}}^{2}\,\mathrm{log}(N)/4$. If *γ* is very small, the degree distribution is not altered by the method of blocking nodes. Thus, we obtain the spreading threshold
  4 $$\lambda  > \frac{\bar{k}}{{\bar{k}}^{2}\,\mathrm{log}(N)/4}\delta =\frac{4\delta }{\bar{k}\,\mathrm{log}(N)}\propto \frac{1}{\bar{k}}$$
  when nodes with a large degree are blocked, these nodes become unreachable. The underlying network is changed, and the average network degree decreases. Therefore, this method increases the spreading threshold, and it becomes more difficult for information to propagate at a large scale.
2. Betweenness
  Betweenness of nodes represents the proportion of the shortest paths across a node. Nodes with large betweenness act as bridges that connect different communities. Through these nodes, information propagates to more indirect nodes. If we block nodes with large betweeness, shortcuts in the network are cut off, and information may be limited to a local region. We sort nodes by their betweenness, and block a proportion *γ* of nodes with the largest betweenness.
3. Clustering coefficient
  Clustering coefficients represent aggregation levels among nodes, and are calculated by counting the number of connected triangles between a node and its neighbors. For nodes with a large clustering coefficient, large connectivity exists among their neighbors. For this method, a proportion *γ* of nodes with the top clustering coefficients are blocked.
4. *k*-core index

The *k*-core index indicates the status of a node according to its location in the network. When a node has a large *k*-core index, it is close to the center of the network. If we block nodes with a large *k*-core index, information may be trapped at the margin of the network. Information cannot reach hub nodes, and does not have a wide impact. For this method, we block a proportion *γ* of nodes with the largest *k*-core index.

### Block diffusion at important edges

We can cut off important edges, instead of blocking nodes. After deleting some edges, the connectivity of the network may be broken. Dynamics are slowed down and the diffusion extent is limited. For this method, a proportion *γ* of edges are removed from the network. Compared with nodes, the number of edges is much larger. Therefore, the value of *γ* in this method should be larger.

1. Strong tie
  The tie strength between two neighboring nodes represents the heterogeneity between their neighborhoods. When nodes from two ends of an edge have more common neighbors, the edge has a large tie strength, and vice versa. It should be investigated whether cutting off the connection of two nodes with a large or small overlap in neighborhood more efficiently restrains information diffusion. We sort all edges by tie strength, and remove a proportion *γ* of edges at the top of the list.
2. Weak tie
  For this method, we cut off a proportion *γ* of edges with the smallest tie strength.
3. Betweenness

We sort edges according to their betweenness and remove a proportion *γ* of edges at the top of the list.

### Distract attention of nodes

Nodes have limited activity, and they do not concentrate on the diffusion of all information. When they contact more than one piece of information, they will choose the information that is of most interest, and consider diffusion actions. Thus, one can create another piece of information to distract node attention and restrain diffusion of the target information. In the SIR model, the spreading probability of information *l*_1_ is defined as *λ*. We introduce information *l*_2_ with spreading probability *λ*_2_, and *l*_2_ meets the public interest. We assume that information *l*_2_ is injected to the network at time *T*. When *t* < *T*, only information *l*_1_ propagates in the system. When *t* > *T*, both pieces of information *l*_1_ and *l*_2_ simultaneously propagate.

We define the densities of susceptible, infected and refractory nodes for information *l*_1_ as *ρ*_1,*s*_(*k*, *t*), *ρ*_1,*i*_(*k*, *t*) and *ρ*_1,*r*_(*k*, *t*), respectively. Similarly, the densities for *l*_2_ are given by *ρ*_2,*s*_(*k*, *t*), *ρ*_2,*i*_(*k*, *t*), and *ρ*_2,*r*_(*k*, *t*). Susceptible nodes may be infected by neighboring spreaders. In the mean-field approximation, a susceptible node at degree *k* connects to an infected node for *l*_1_ at degree *k*′ with probability $P({i}_{1},k^{\prime} |{s}_{1},k)\approx {\rho }_{1,i}(k^{\prime} ,t)$.

**Figure 1:**
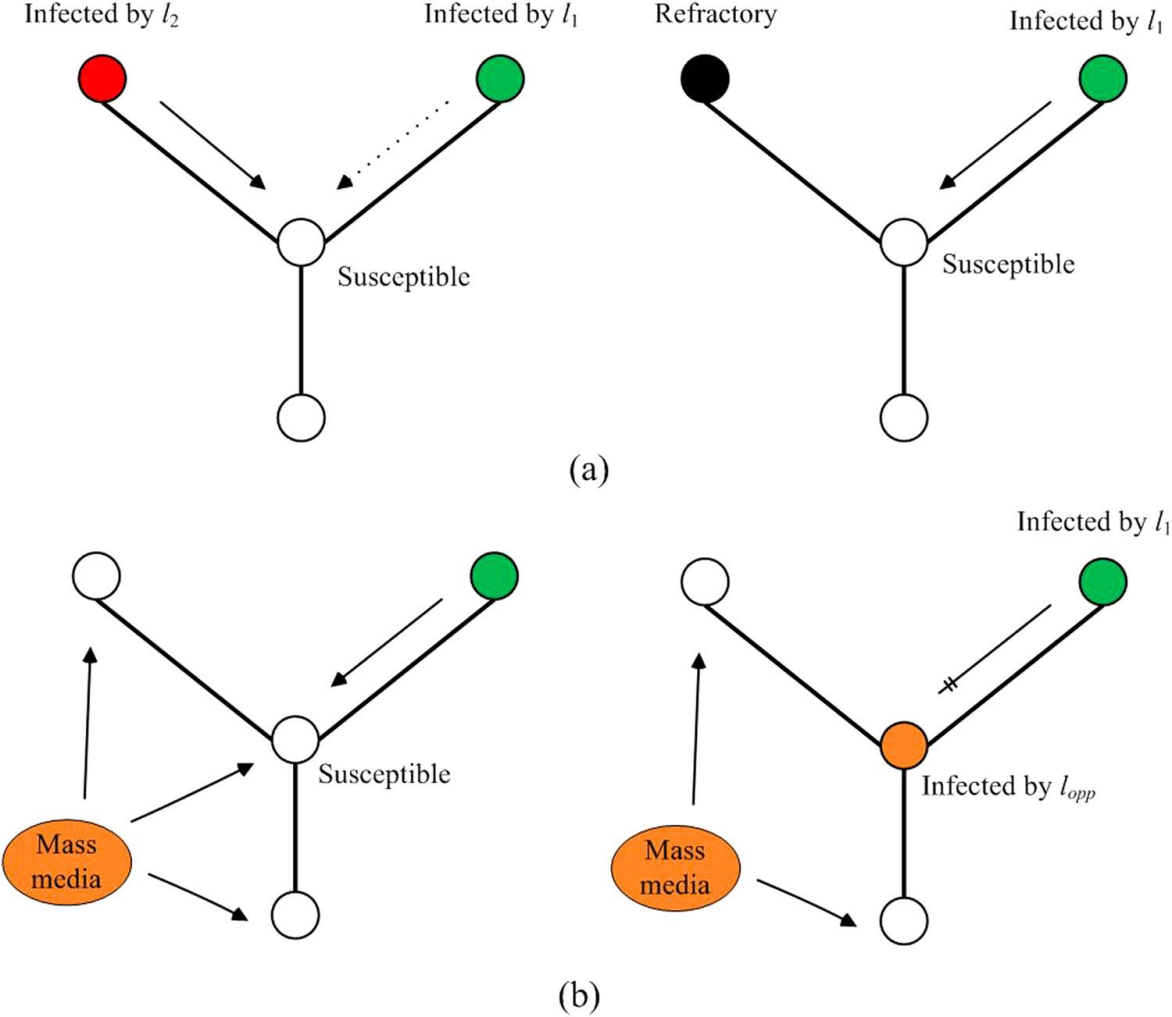
Graphical illustration for dynamic spread of both pieces of information in different methods. (**a**) Distract attention of nodes. When a susceptible node in the left plot receives information *l*_1_ and *l*_2_ at the same time, it preferentially selects information *l*_2_. Therefore, the transmission from infected nodes of *l*_1_ to the susceptible node is temporarily suspended. In the right plot, when spreaders of *l*_2_ become refractory, the transmission of *l*_1_ is recovered. (**b**) Propagate opposite information by mass media. In the left plot, information *l*_*opp*_ from mass media acts on all susceptible nodes. In the right plot, if a node is infected by the opposite information, the transmission from spreaders of *l*_1_ to the node is permanently cut off.

With the simultaneous diffusion of the information, the dynamics are governed by the following rules. The illustration for the diffusion of both pieces of information can be seen in Fig. 1a.

- When a susceptible node with degree *k* for information *l*_2_ contacts an infected neighbor of *l*_2_, the node may be infected by *l*_2_. Considering the influence of network topology, the probability of being infected by *k* neighboring spreaders during each time is defined as *P*(*i*_2_, *k*). Analogously to ref. 9, *P*(*i*_2_, *k*) is obtained by
  5 $$P({i}_{2},k)=1-\mathop{\mathrm{lim}}\limits_{{\rm{\Delta }}t\to 0}{(1-{\lambda }_{2}{\rm{\Delta }}t)}^{k/{\rm{\Delta }}t}=1-{e}^{-{\lambda }_{2}k}$$
  where *λ*_2_ is the spreading rate for *l*_2_. Here, we only consider discrete time steps, and the probability at each time is approached by $P({i}_{2},k)={\lambda }_{2}k$.
  For the susceptible node, the average density of neighboring spreaders for *l*_2_ is written as
  6 $$\rho ({i}_{2}|{s}_{2},k)=\sum _{k^{\prime} }P(k^{\prime} |k){\rho }_{2,i}(k^{\prime} ,t)$$
  Therefore, a susceptible node with degree *k* may be infected by *l*_2_ at time *t* with probability
  7 $${P}_{{s}_{2}\to {i}_{2}}(k,t)=P({i}_{2},k)\rho ({i}_{2}|{s}_{2},k)={\lambda }_{2}k\sum _{k^{\prime} }P(k^{\prime} |k){\rho }_{2,i}(k^{\prime} ,t)$$
- When a susceptible node with degree *k* for information *l*_1_ contacts an infected node of *l*_1_, the node may be infected by *l*_1_. If the susceptible node does not receive information *l*_2_, the probability of being infected by *k* neighboring spreaders is *P*(*i*_1_, *k*) = *λk*; otherwise, the probability of being infected by *l*_1_ is 0.
  For the susceptible node, the average density of neighboring spreaders for *l*_1_ that are non-spreaders for *l*_2_ is written as
  8 $$\rho ({i}_{1},{\rm{not}}\,{i}_{2}|{s}_{1},k)=\sum _{k^{\prime} }P(k^{\prime} |k){\rho }_{1,i}(k^{\prime} ,t)(1-{\rho }_{2,i}(k^{\prime} ,t))$$
  Therefore, a susceptible node with degree *k* may be infected by *l*_1_ at time *t* with probability
  9 $${P}_{{s}_{1}\to {i}_{1}}(k,t)=P({i}_{1},k)\rho ({i}_{1},\,{\rm{not}}{i}_{2}|{s}_{1},k)=\lambda k\sum _{k^{\prime} }P(k^{\prime} |k){\rho }_{1,i}(k^{\prime} ,t)(1-{\rho }_{2,i}(k^{\prime} ,t))$$
- Each time, spreaders of information *l*_1_ and *l*_2_ become refractory with probability *δ*.

Overall, in the diffusion process, information *l*_2_ that is injected at time *T* starts spreading over the network during the transient phase of information *l*_1_, and it exerts an inhibitory effect on *l*_1_ after *T*.

When a node simultaneously contacts information *l*_1_ and *l*_2_, *l*_2_ is preferentially adopted. Therefore, the probability that a susceptible node is infected by spreaders for *l*_2_ is the same as that in the SIR model; therefore, the transition of densities for *l*_2_ agrees with Eq. (1).

Now, we study the diffusion of information *l*_1_. We obtain the transition of *ρ*_1,*s*_(*k*, *t*), *ρ*_1,*i*_(*k*, *t*), and *ρ*_1,*r*_(*k*, *t*) from the model.

10 $$\begin{array}{c}\frac{\partial {\rho }_{1,s}(k,t)}{\partial t}=-\lambda k{\rho }_{1,s}(k,t)\sum _{k^{\prime} }P(k^{\prime} |k){\rho }_{1,i}(k^{\prime} ,t)(1-{\rho }_{2,i}(k^{\prime} ,t))\\ \frac{\partial {\rho }_{1,i}(k,t)}{\partial t}=\lambda k{\rho }_{1,s}(k,t)\sum _{{k}^{\text{'}}}P(k^{\prime} |k){\rho }_{1,i}(k^{\prime} ,t)(1-{\rho }_{2,i}(k^{\prime} ,t))-\delta {\rho }_{1,i}(k,t)\\ \frac{\partial {\rho }_{1,r}(k,t)}{\partial t}=\delta {\rho }_{1,i}(k,t)\end{array}$$

When *t* < *T*, it holds true that *ρ*_2,*i*_(*k*′, *t*) = 0, and the diffusion of *l*_1_ is the same as the SIR model. If *T* > 0, the spreading threshold for information *l*_1_ remains unchanged under the action of *l*_2_. Then, we consider the special case *T* = 0, i.e., information starts to propagate at the beginning of the dynamics. We integrate the first equation of Eq. (10) from time 0 to time *t*, and obtain

11 $${\rho }_{1,s}(k,t)={\rho }_{1,s}(k,0){e}^{-\lambda k\varphi (t)}$$

where *ρ*_1,*s*_(*k*, 0) is the initial density of susceptible nodes for information *l*_1_, and it yields *ρ*_1,*s*_(*k*, 0) ≈ 1. The auxiliary function *ϕ*(*t*) is expressed as

12 $$\begin{array}{rcl}\varphi (t) & = & \sum _{{k}^{\text{'}}}P(k^{\prime} |k){\int }_{0}^{t}{\rho }_{1,i}(k^{\prime} ,t^{\prime} )(1-{\rho }_{2,i}(k^{\prime} ,t^{\prime} ))dt^{\prime} \\  & = & \frac{1}{\bar{k}}\sum _{k^{\prime} }k^{\prime} P(k^{\prime} ){\int }_{0}^{t}{\rho }_{1,i}(k^{\prime} ,t^{\prime} )(1-{\rho }_{2,i}(k^{\prime} ,t^{\prime} ))dt^{\prime} \end{array}$$

Multiplying both sides of the second equation of Eq. (10) by $kP(k)/\bar{k}$, and summing over different *k*, we have the following expression.

13 $$\begin{array}{c}\frac{1}{\bar{k}}\sum _{k}kP(k)\frac{\partial {\rho }_{1,i}(k,t)}{\partial t}=\,\frac{\lambda }{\bar{k}}\sum _{k}{k}^{2}P(k){\rho }_{1,s}(k,t)\sum _{{k}^{\text{'}}}\frac{k^{\prime} P(k^{\prime} )}{\bar{k}}{\rho }_{1,i}(k^{\prime} ,t)(1-{\rho }_{2,i}(k^{\prime} ,t))-\frac{\delta }{\bar{k}}\sum _{k=0}^{+\infty }kP(k){\rho }_{1,i}(k,t)\\ \phantom{\rule{5em}{0ex}}\qquad \quad \,\,\,=\,\frac{\lambda }{\bar{k}}\sum _{k}{k}^{2}P(k){\rho }_{1,s}(k,t)\frac{d\varphi (t)}{dt}-\frac{\delta }{\bar{k}}\sum _{k=0}^{+\infty }kP(k){\rho }_{1,i}(k,t)\end{array}$$

We integrate Eq. (13) by *t*, and obtain

14 $$\frac{1}{\bar{k}}\sum _{k}kP(k){\rho }_{1,i}(k,t)=\frac{\lambda }{\bar{k}}\sum _{k}{k}^{2}P(k){\int }_{0}^{t}{e}^{-\lambda k\varphi (t^{\prime} )}\frac{d\varphi (t^{\prime} )}{dt^{\prime} }dt^{\prime} -\frac{\delta }{\bar{k}}\sum _{k=0}^{+\infty }kP(k){\int }_{0}^{t}{\rho }_{1,i}(k,t^{\prime} )dt^{\prime} $$

Noting that when *t* → +∞, *ρ*_1,*i*_(*k*, *t*) = 0, after some elementary manipulations, Eq. (14) becomes

15 $$0=\frac{1}{\bar{k}}\sum _{k}kP(k)(1-{e}^{-\lambda k\varphi (\infty )})-\frac{\delta }{\bar{k}}\sum _{k=0}^{+\infty }kP(k){\int }_{0}^{+\infty }{\rho }_{1,i}(k,t^{\prime} )dt^{\prime} $$

We focus on the second part of the right side of Eq. (15). When information *l*_2_ can propagate in the network, i.e., ${\lambda }_{2} > \bar{k}\delta /\bar{{k}^{2}}$, it holds true that 1 − *ρ*_2,*i*_(*k*,* t*) < 1. Therefore, we have

16 $$\varphi (\infty )=\frac{1}{\bar{k}}\sum _{{k}^{\text{'}}}k^{\prime} P(k^{\prime} ){\int }_{0}^{+\infty }{\rho }_{1,i}(k^{\prime} ,t^{\prime} )(1-{\rho }_{2,i}(k^{\prime} ,t^{\prime} ))dt^{\prime}  < \frac{1}{\bar{k}}\sum _{k^{\prime} }k^{\prime} P(k^{\prime} ){\int }_{0}^{+\infty }{\rho }_{1,i}(k^{\prime} ,t^{\prime} )dt^{\prime} $$

We assume that

17 $$\frac{1}{\bar{k}}\sum _{k^{\prime} }k^{\prime} P(k^{\prime} ){\int }_{0}^{+\infty }{\rho }_{1,i}(k^{\prime} ,t^{\prime} )dt^{\prime} =\beta \varphi (\infty )$$

where *β* is a constant with *β* > 1. Around the threshold, *ϕ*(∞) is small, so *e* ^−*λkϕ*(∞) ^→ 1. Inserting Eq. (17) into Eq. (15) and making the Taylor expansion of* e* ^−*λkϕ*(∞)^,we obtain

18 $$0=\frac{1}{\bar{k}}\sum _{k}(\lambda {k}^{2}P(k)\varphi (\infty )-{\lambda }^{2}{k}^{3}P(k){\varphi }^{2}(\infty ))+o({\varphi }^{2}(\infty ))-\beta \delta \varphi (\infty )$$

We calculate *ϕ*(∞) from Eq. (18)

19 $$\varphi (\infty )=\frac{\lambda \overline{{k}^{2}}/\bar{k}-\beta \delta }{{\lambda }^{2}\overline{{k}^{3}}/\bar{k}}$$

When information *l*_1_ can propagate, it should hold true that *ϕ*(∞) > 0. Therefore, we obtain the spreading threshold for *l*_1_

20 $$\lambda  > \frac{\beta \delta \bar{k}}{\overline{{k}^{2}}}$$

As *β* > 1, the spreading threshold increases. Therefore, when *T* = 0, the method of distracting node attention changes both the spreading threshold and diffusion extent.

### Propagate opposite information by mass media

We can utilize mass media to propagate information *l*_*opp*_ that has the opposite content with current information *l*_1_, and to restrain the diffusion of *l*_1_. For instance, website administrators may clarify the truth to restrain rumor diffusion in online networks. The authorities create positive information and propagate it. The information is often located in conspicuous places of the website, such that it can easily be seen by all users. When users read information that originates from mass media, they are likely to accept the information. If they are convinced by the truth, they will no longer be infected by the rumor. Assume that the opposite information *l*_*opp*_ is injected to the network at time *T*, and nodes are infected by it with probability *λ*_*opp*_. The model evolves following the rules below. Figure 1b illustrates the dynamic diffusion.

- A susceptible node with degree *k* may be infected by *l*_1_ at time *t* with probability
  21 $${P}_{{s}_{1}\to {i}_{1}}(k,t)=\lambda k\sum _{k^{\prime} }P(k^{\prime} |k){\rho }_{1,i}(k^{\prime} ,t)$$
- Each time, infected nodes of *l*_1_ become refractory with probability *δ*.
- Information *l*_*opp*_ is injected to the network at time *T*. It infects all susceptible nodes with probability *λ*_*opp*_. In that case, if a node was previously in the susceptible state, it will never be infected by *l*_1_.

The density of susceptible nodes for *l*_1_ decreases by the inhibitory action of *l*_*opp*_. We obtain the transition of densities in different states for information *l*_1_ when *t* > *T*.

22 $$\begin{array}{c}\frac{\partial {\rho }_{1,s}(k,t)}{\partial t}=-\lambda k{\rho }_{1,s}(k,t)\sum _{k^{\prime} =0}^{+\infty }P(k^{\prime} |k){\rho }_{1,i}(k^{\prime} ,t)-{\lambda }_{opp}{\rho }_{1,s}(k,t)\\ \frac{\partial {\rho }_{1,i}(k,t)}{\partial t}=\lambda k{\rho }_{1,s}(k,t)\sum _{k^{\prime} =0}^{+\infty }P(k^{\prime} |k){\rho }_{1,i}(k^{\prime} ,t)-\delta {\rho }_{1,i}(k,t)\\ \frac{\partial {\rho }_{1,r}(k,t)}{\partial t}=\delta {\rho }_{1,i}(k,t)\end{array}$$

Obviously, the diffusion process is divided into two stages, i.e., *t* < *T* and *t* > *T*. For *t* < *T*, the evolution is the same as the traditional SIR model. Now we consider the case *t* > *T*. After time *T*, it holds true that ${\rho }_{1,s}(k,T)+{\rho }_{1,i}(k,T)+{\rho }_{1,r}(k,T)\ne 1$. If *T* is large, it is easy to infer from the second equation of Eq. (22) that the spreading threshold is not changed by mass media. If *T* is small, the influence of mass media cannot be neglected. The system is governed by two dynamics: the diffusion of information *l*_1_ and its opposite edition *l*_*opp*_. These two pieces of information have different time scales of diffusion. Compared with information *l*_1_, the dynamics of *l*_*opp*_ are much faster because it is published by mass media and can simultaneously act on all nodes. Therefore, when *T* is sufficiently small, in the early stage of the diffusion process for *l*_1_, the initial condition is ${\rho }_{1,i}(k,t)\approx 0$ at *t* = *T* + Δ*t*, but the initial density of susceptible nodes *ρ*_1,*s*_(*k*,*t*) does not approach 1. Therefore, the spreading threshold for *l*_1_ increases. We conclude that for small *T*, this method not only affects the threshold for the diffusion of *l*_1_, but it also changes the diffusion extent.

## Results

We conducted Monte-Carlo simulations to investigate the effects of the aforementioned four methods. Initially, a node is selected at random, and it is set in the infected state, whereas all the other nodes are susceptible. The system size is defined as *N*. Simulations are synchronously performed. At each time step, all infected nodes contact their neighbors, and persuade them to participate in diffusion. Then, infected nodes may simultaneously become refractory. As these restraining methods have different scales and action mechanisms, we cannot depict the results together in the same parameter space. However, we can compare the effects of these methods on the spreading threshold, relaxation speed and other properties as well as determine the optimal restraining method in different situations. It should be noted that the methods of removing nodes and edges increase the cost, because the network connectivity is destroyed. Barabasi-Albert scale-free networks and a real social network are used as interaction topology. The average degree of scale-free networks is $\bar{k}=4$. The social network was collected from the Sina micro-blog (www.weibo.com), which is one of the most popular online social networks in China. The network contains 5906 nodes. The average degree is $\bar{k}=4.5008$, and the average clustering coefficient is 0.0772. First, we separately studied the effects of the four restraining methods, and then we compared their mechanisms together.

### Block nodes

**Figure 2:**
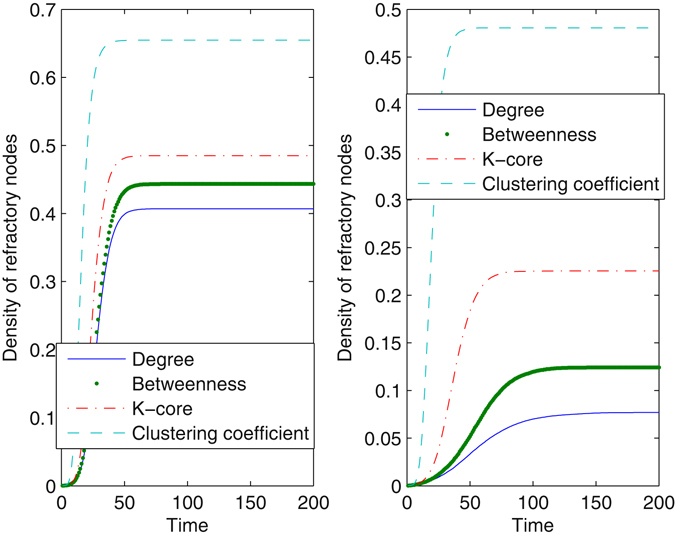
Density of refractory nodes as a function of time with the method of blocking different nodes. The underlying network is a scale-free network, *N* = 2500, *λ* = 0.2, and *δ* = 0.2. In the left plot, the proportion of nodes that are blocked is *γ* = 0.01. In the right plot, *γ* is set at 0.05. Every plot is an average of 100 different simulations.

Figure 2 shows the time evolution for the density of refractory nodes with the method of blocking different nodes in a scale-free network. The method of blocking nodes with large degrees takes effect most evidently, whereas blocking nodes with large cluster coefficients seems ineffective for preventing information diffusion. Increasing the proportion of nodes that are blocked clearly decreases the number of refractory nodes, reducing the impact of the information. However, even when *γ* = 0.05, information still succeeds in propagating in the network, as a result of high connectivity in the network. When *γ* = 0.05, very few nodes are infected from the method of blocking nodes with large degrees. The effect of betweenness is larger than the *k*-core, especially when *γ* increases. The method restrains information diffusion from the beginning of the evolutionary process until the system stabilizes. To further understand the essential mechanism of the method, we observed the impact of the method of blocking different nodes on network connectivity. When nodes are blocked according to their betweenness, the average degree of the network drops to 3.1016 for *γ* = 0.01 and 2.1856 for *γ* = 0.05. When the method regarding clustering coefficients is used, the average degree is 3.9552 for *γ* = 0.01 and 3.1680 for *γ* = 0.05. Obviously, blocking nodes at large betweenness increases the loss of network connectivity. With more extensive damage to network connectivity, information can be seriously limited.

**Figure 3:**
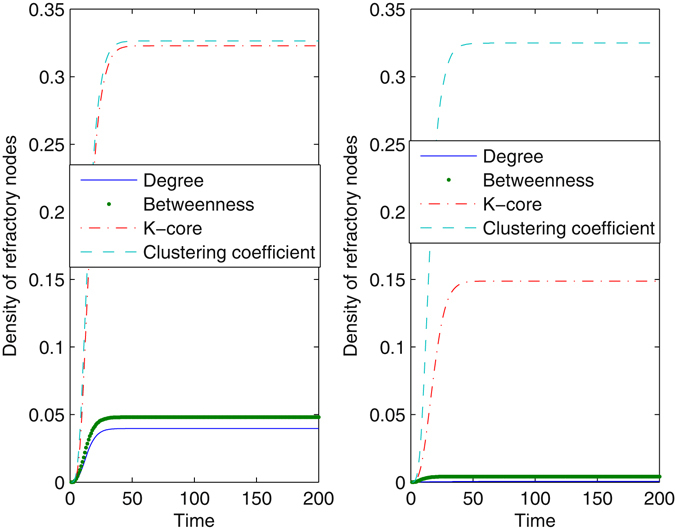
Density of refractory nodes as a function of time with the method of blocking different nodes. The underlying network is the real network, *λ* = 0.2, and *δ* = 0.2. In the left plot, the proportion *γ* is 0.01. In the right plot, *γ* is set at 0.05. Every plot is an average of 100 different simulations.

Figure 3 shows the evolution in the real network. The results are analogous to those in a scale-free network. The effect of the degree-dependent method almost approaches the betweenness-dependent method, especially when *γ* = 0.05. However, the difference between the effect of *k*-core and betweenness is enlarged in the real network. Dramatically, the final density of refractory nodes remains nearly unchanged under the action of the method regarding clustering coefficients, when the proportion of blocked nodes increases. We observed that the average clustering coefficient in the real network is much larger than that in a scale-free network. Comparing Fig. 3 with Fig. 2, the extent of information diffusion drops in the real network, as a result of a more heterogeneous degree distribution. We normalized the distribution to [0, 1] and quantize it with equal intervals. Next, we calculated the Gini coefficient of the distribution^34^. The Gini coefficient of node degrees in a scale-free network is 0.9645, whereas that in the real network is 0.9825. If some nodes in the shortcuts are blocked, the network may be divided into several unconnected communities, such that information diffusion is trapped in a single community. For *γ* = 0.05, if nodes with large degrees or betweenness are blocked, information cannot propagate in the real network.

**Figure 4:**
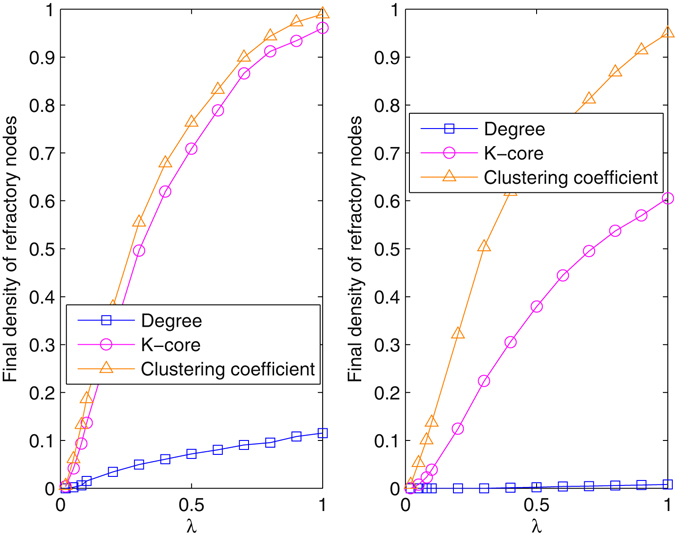
Final density of refractory nodes versus *λ*. The underlying network is the real network, and *δ* = 0.2. (**a**) *γ* = 0.01; (**b**) *γ* = 0.05. The results are averaged over 100 realization.

We investigated the impact of the method on the spreading threshold, as shown in Fig. 4. As the effect of the betweenness-dependent method is close to that of the degree-dependent method, the result is not shown here when blocking nodes with large betweenness. Obviously, blocking nodes changes the threshold of *λ*, which agrees with analytical results. By increasing the restraining proportion *γ*, the spreading threshold is enlarged especially for the degree-dependent method, and some nodes are always blocked from the information even when *λ* = 1. With the method regarding *k*-core and clustering coefficients, when *γ* is small, the threshold is almost 0, implying that it is easy for information to propagate in the real network. In the right plot, when *γ* is increased, a small threshold is observed for the core-dependent method, but the threshold remains 0 when blocking nodes with large clustering coefficients. Therefore, the method regarding clustering coefficients hardly affects the threshold for information diffusion.

### Block edges

**Figure 5:**
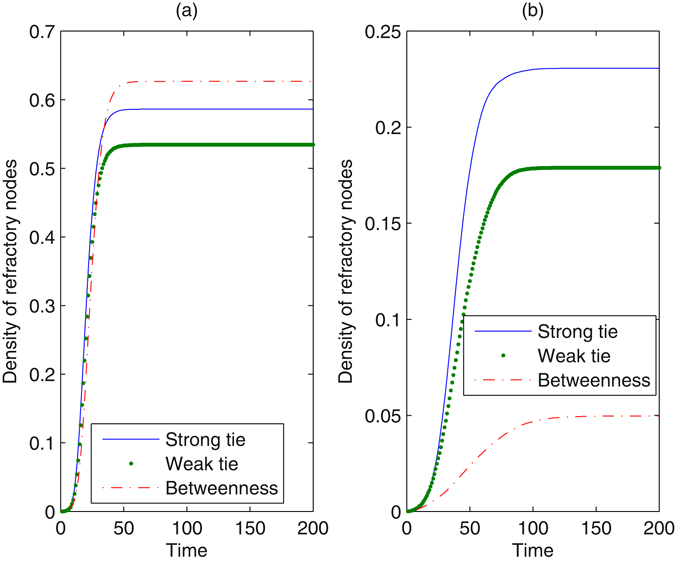
Density of refractory nodes with the method of blocking edges. The underlying network is a scale-free network, *λ* = 0.2, and *δ* = 0.2. In the left plot, the proportion of edges that are blocked is *γ* = 0.1. In the right plot, *γ* is set at 0.4. Every plot is an average of 100 different simulations.

**Figure 6:**
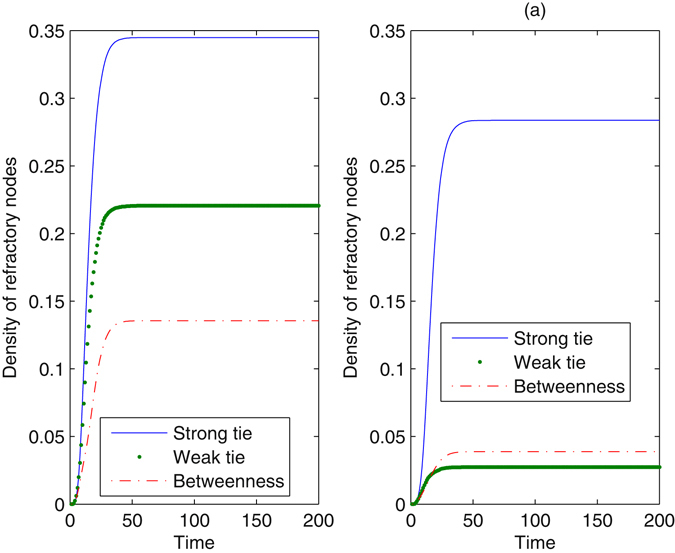
Density of refractory nodes with the method of blocking edges. The underlying network is the real network, *λ* = 0.2, and *δ* = 0.2. In the left plot, *γ* = 0.1, and in the right plot, *γ* = 0.4. Every plot is an average of 100 different simulations.

Figure 5 illustrates the effect of blocking diffusion at different edges in a scale-free network. From this figure, the method of blocking weak ties achieves a better performance in all situations than blocking strong ties, meaning that weak ties act as bridges to connect isolated groups together and deleting weak ties can trap information in local regions. However, blocking these edges cannot completely prevent information diffusion. Even if the proportion of these edges that are blocked is 0.4, more than 17% of nodes are affected by the information. The method of blocking edges with large betweenness takes effect depending on the proportion *γ*. When *γ* is small, blocking edges with large betweenness cannot obviously prevent the occurrence of refractory nodes; however, for large *γ*, betweenness becomes the most effective feature. Because there are many shortcuts in the scale-free network, information can still propagate at a large scale through remaining shortcuts unless sufficient edges are removed. As shown in Fig. 6, because there is large heterogeneity in the edge distribution of the real network, more nodes can be prevented from information in the network than in a scale-free network except for the method of blocking strong ties. This result is similar to that shown in Fig. 3. Betweenness remains an important feature for restraining diffusion, despite *γ*. We also observed that the method of blocking edges has little effect on the spreading threshold, except for the case of a small spreading threshold from the weak tie-dependent method with large *γ*.

### Distract the attention of nodes

**Figure 7:**
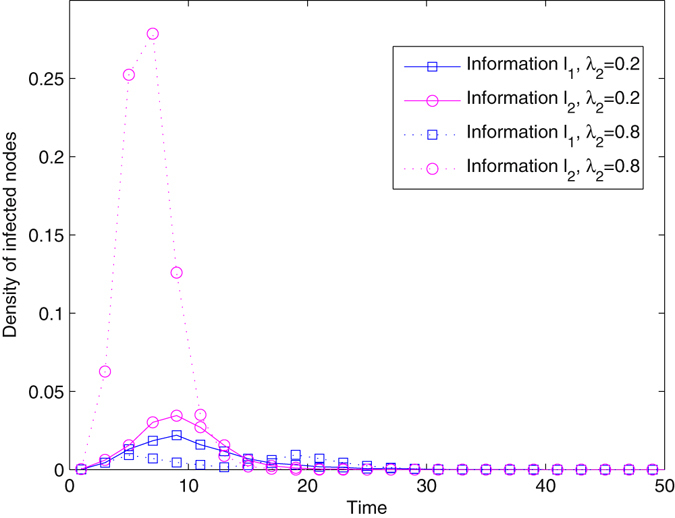
Density of infected nodes for two pieces of information with the method of distracting node attention. The real network mediates the diffusion, *T* = 0, *λ* = 0.2, and *δ* = 0.5.

We introduced information *l*_2_ that is more attractive to all nodes, and subsequently studied its restraining action on the diffusion of *l*_1_. Figure 7 shows the time evolution of infected nodes for two pieces of information. *l*_2_ is injected into the network at the beginning, i.e., *T* = 0, and therefore, *l*_1_ is influenced as soon as it propagates. When the spreading probability of information *l*_2_ is small, the evolutionary trend of the density of infected nodes for *l*_1_ is similar to *l*_2_, implying that the restraining action is generated all along with the diffusion of *l*_1_. The densities of infected nodes for *l*_1_ and *l*_2_ increase at first, and then peak at almost the same time. Finally, all infected nodes for *l*_1_ and *l*_2_ quickly become extinct. The maximal density for *l*_1_ is not far from that for *l*_2_, meaning the impact is not significant. When *λ*_2_ is large, the peak for *l*_2_ is greatly enhanced. It is dramatic that the density of infected nodes for *l*_1_ has two peaks. When the density of spreaders for *l*_2_ drops to a low level, more infected nodes for *l*_1_ occur and their density increases. Therefore, the restraining action plays a role in the initial phase of diffusion. When information *l*_2_ stops propagating, the diffusion of *l*_1_ is recovered. However, the maximal density for *l*_1_ declines severely, implying that a strong restraining effect has occurred. In addition, the method of distracting node attention accelerates the diffusion process. Within less than 30 time steps, all infected nodes become refractory, leading to a short relaxation time of the system.

**Figure 8:**
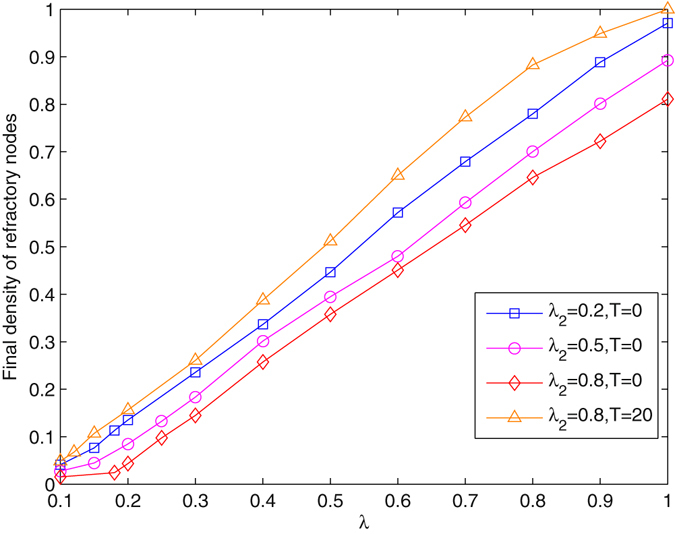
Final density of refractory nodes for information *l*_1_ in the real network with the method of distracting node attention, *δ* = 0.5.

Figure 8 shows the final density of refractory nodes for information *l*_1_ under different actions of *l*_2_. When information *l*_2_ propagates at the beginning, the spreading threshold of *l*_1_ is changed. For *T* = 0, larger spreading probability *λ*_2_ increases the threshold of *l*_1_, and decreases the final density of refractory nodes. However, if the diffusion of information *l*_2_ is delayed, the threshold of *l*_1_ remains nearly unchanged, coinciding with the analysis. Therefore, the spreading threshold is sensitive to the action time of this method. Especially, when *T* = 20, more nodes become refractory even with large *λ*_2_. Unlike Fig. 4, the final density for *l*_1_ approximately increases linearly with *λ* beyond the threshold, and it will reach a large value even if the spreading capacity of *l*_2_ is strong. Because a large proportion of nodes may be infected during the diffusion process, the method cannot efficiently prevent the diffusion of information that has large spreading probability.

### Propagate opposite information by mass media

**Figure 9:**
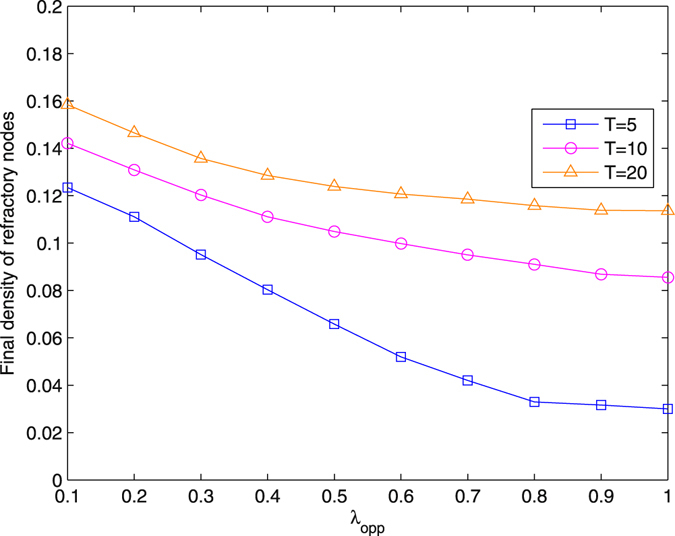
Final density of refractory nodes for information *l*_1_ in the real network with the method of propagating opposite information, *λ* = 0.2, and *δ* = 0.5.

Figure 9 demonstrates the restraining result for the method of propagating opposite information by mass media. Compared with the method of distracting node attention, this method has a more distinct effect. Increasing the spreading probability of opposite information *λ*_*opp*_, the diffusion of *l*_1_ is restrained and the final density of refractory nodes decreases especially when *T* is smaller. The final density decreases for *λ*_*opp*_ < 0.8, but it gradually stabilizes for *λ*_*opp*_ > 0.8. Therefore, it is not economical to promote the spreading probability for mass media beyond 0.8. When *T* = 5, the threshold for *l*_1_ is changed with *λ*_*opp*_, which agrees with the analysis. However, when *T* > 10, the threshold becomes independent of *λ*_*opp*_, and the decrease for the density of refractory nodes is not marked as *λ*_*opp*_ increases. Compared with the spreading probability of opposite information, the time when it is injected plays a far more important role in restraining *l*_1_. Even if the spreading probability *λ* of *l*_1_ is increased, *l*_1_ can be effectively restrained by mass media on the condition that mass media work as early as possible. The effect is different from the method of distracting node attention.

### Comparison and combination

From the left plot of Fig. 3 and right plot of Fig. 6, the method of blocking nodes with large betweenness for *γ* = 0.01 has a similar restraining effect to that of blocking edges with large betweenness for *γ* = 0.4. By blocking edges for *γ* = 0.4, 80.41% nodes in the network are involved with these removed edges; however, by blocking nodes, the proportion of involved nodes is 1%. On the one hand, more effort should be made to restrain the diffusion by the method of blocking edges, and the method of blocking nodes is more effective. On the other hand, when nodes are blocked, all the edges that link to these nodes are invalid, destroying the network connectivity. After blocking nodes with large betweenness, the average degree of the network is 2.4840, whereas after blocking edge, the average degree is 2.7005. Therefore, the method of blocking nodes causes a larger loss of network connectivity.

With respect to injecting another piece of information, the method of distracting node attention can be regarded as an internal field, and propagating opposite information refers to an external field. These two methods exert an obvious effect in the early stage of diffusion. Especially for distracting node attention, only when *T* is approximately 0, the spreading threshold is changed, and the final density of refractory nodes decreases. The method of propagating opposite information is more effective because it is induced by mass media.

**Figure 10:**
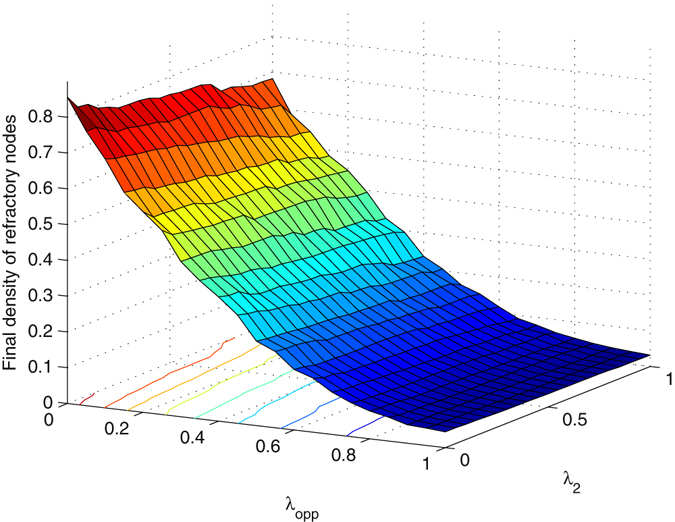
Final density of refractory nodes for information *l*_1_ versus *λ*_2_ and *λ*_*opp*_ when the methods of distracting node attention and propagating opposite information are included. *λ* = 0.8, *δ* = 0.5 and *T* = 2.

We integrated multiple methods to restrain information diffusion and investigated the coherent action of these methods. Figure 10 illustrates the joint effect of methods of distracting node attention and propagating opposite information by mass media. Here, we considered that the target information has large spreading probability *λ*, and studied whether the methods are sufficient to restrain it. In Fig. 10, mass media considerably reduce the final diffusion extent of the information. Although the method of distracting node attention makes the final refractory density decrease, the target information is not effectively limited. The coherent effect of the two methods is mainly determined by mass media. Contours of the curved surface are nearly parallel to the axis of *λ*_2_, implying that increasing *λ*_*opp*_ has a great advantage over *λ*_2_. Therefore, for information with large spreading probability, a better approach to restrain diffusion is to propagate opposite information by mass media, although more cost may be required.

**Figure 11:**
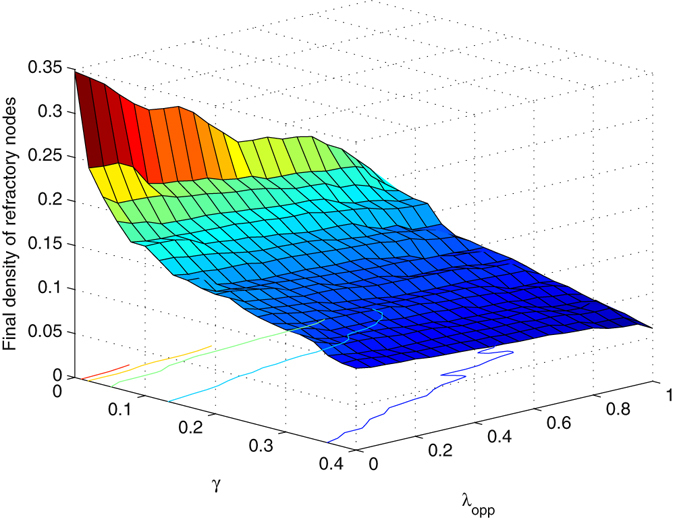
Final density of refractory nodes for information *l*_1_ versus *γ* and *λ*_*opp*_ when the methods of blocking nodes and propagating opposite information are included. *λ* = 0.2, *δ* = 0.2 and *T* = 10.

We investigated the combination of blocking nodes with large degrees and propagating opposite information. Here, we considered that the target information is detected late after the early stage, and investigated whether these remedial measures are effective when they do not work early. To achieve the same baseline, we did not block nodes at the beginning; instead, we blocked them at *t* = *T*. From Fig. 11, both methods reduce the final density of refractory nodes. The contours of the curved surface are not parallel to the coordinate axis; instead, they correlate with both *γ* and *λ*_*opp*_, demonstrating the joint effect of the two methods. Large *γ* restrains the diffusion, and large *λ*_*opp*_ further lessens the refractory nodes. Moreover, blocking nodes has a stronger influence and the diffusion extent has a dramatic drop with this method, but propagating opposite information cannot limit the diffusion at a low level. Therefore, if the method is not adopted in a timely manner, the only effective choice is to block nodes at large degrees.

## Conclusions

To restrain information diffusion, we may remove nodes and community bridges to break network connectivity and trap information in local regions. Substantial effort should be made to block many elements in a large-scale network. Meanwhile, we can also impose a field to the network, and affect interactions among nodes. The field may work from the outside or inside of the network. In this paper, we presented four methods of restraining information diffusion. These methods belong to two categories: changing the network structure and changing node behavior. Based on the epidemic model, we analyzed the spreading threshold of the target information with the mean-field approach, and investigated the effects on the final diffuse extent with different conditions.

The results indicate that the methods restrain information diffusion and change the spreading threshold in defined conditions. Blocking nodes with large degrees or betweenness effectively prevents diffusion, and the method may even completely limit information to local regions. Blocking edges with large betweenness has a distinct effect in the real network. The method of blocking nodes is more efficient and economical than blocking edges. Distracting node attention restrains the diffusion only when the method is adopted at the beginning, and it is only available for information with small spreading capabilities. The effect of propagating opposite information is more determined by the action time than the action intensity. Different situations require the selection of optimal methods that lead to a compromise between effectiveness and cost. For information with small spreading probability, distracting node attention can restrain diffusion. However, for information with large spreading probability, the better approach is to propagate opposite information. If the remedial measure works after the early stage of information diffusion, the only effective method is to block nodes with large degrees or betweenness.

In the present work, we assume that the imposed field has the same influence on each node. However, nodes may have different behaviors under identical measures. In the future, we will investigate how node behaviors are affected in detail and study the influence of heterogeneous spreading capabilities.
